# The complete mitochondrial genome of *Triplophysa scleroptera* and its phylogenetic placement among related nemacheilid taxa

**DOI:** 10.1080/23802359.2026.2668246

**Published:** 2026-05-10

**Authors:** Fei Li, Zengxiang Guo, Qiang Hu, Yan Pan, Weijun Wu, Jie Chen

**Affiliations:** a College of Fishery Economics, Tangshan Maritime Institute, Tangshan, China; b Tangshan Fengnan District Bureau of Agriculture and Rural Affairs, Tangshan, China; c The Fifth Middle School of Songyang County, Lishui, China; d Qingyuan County Bureau of Agriculture and Rural Affairs, Lishui, China; e Industrial College of Traditional Chinese Medicine and Health, Lishui University, Lishui, China

**Keywords:** Mitogenome assembly, phylogenetic reconstruction, plateau loach

## Abstract

We report the complete mitochondrial genome of *Triplophysa scleroptera* (Cypriniformes: Nemacheilidae), an endemic plateau loach from the upper Yellow River, China. The circular mitogenome is 16,572 bp in length and comprises 13 protein-coding genes (PCGs), 22 tRNA genes, two rRNA genes, and a control region, with a typical teleost gene arrangement and A + T-biased base composition. Phylogenetic analysis based on the concatenated 13 PCGs from *T. scleroptera* and related nemacheilids showed that *T. scleroptera* clustered within the sampled *Triplophysa* taxa and was sister to the clade formed by *T. tenuis* and *T. bombifrons*.

## Introduction

Nemacheilidae (stone loaches) is a diverse family of small benthic cypriniform fishes inhabiting streams and rivers across Eurasia (Rashid et al. 2025). Within this family, the genus *Triplophysa* is the most species-rich lineage, and many species are endemic to the Qinghai–Tibet Plateau and adjacent drainages, where they have evolved adaptations to high-altitude and low-temperature environments (Wang et al. 2016; Chen et al. 2019). However, rapid diversification and conservative external morphology have led to taxonomic uncertainty in several *Triplophysa* complexes, and additional molecular data are needed to clarify their relationships (Chen et al. 2019).

Mitochondrial DNA markers, particularly complete mitochondrial genomes, provide abundant phylogenetically informative characters and have been widely used to resolve species boundaries and infer evolutionary history in nemacheilid loaches (Zhao et al. 2023; Xu et al. 2024). *Triplophysa scleroptera* (Herzenstein, 1888) is an endemic loach mainly distributed in Qinghai Lake and the upper reaches of the Yellow River, where it is an important component of local benthic communities and plateau aquatic ecosystems (Li et al. 2023; Feng et al. 2025). Ecological and life-history traits of *T. scleroptera* have been described, and a chromosome-level nuclear genome assembly and basic mitochondrial genome information are already available for this species (Li et al. 2023; Feng et al. 2025). Nevertheless, a dedicated mitogenome-centered characterization and phylogenetic analysis remain useful for comparative mitogenomics and evolutionary assessment within *Triplophysa*.

Here, we assembled and annotated the complete mitochondrial genome of *T. scleroptera*, described its basic genomic features, and reconstructed a maximum-likelihood phylogeny based on 13 protein-coding genes (PCGs) to assess the phylogenetic position of *T. scleroptera* among sampled *Triplophysa* taxa.

## Materials and methods

**Figure 1.**
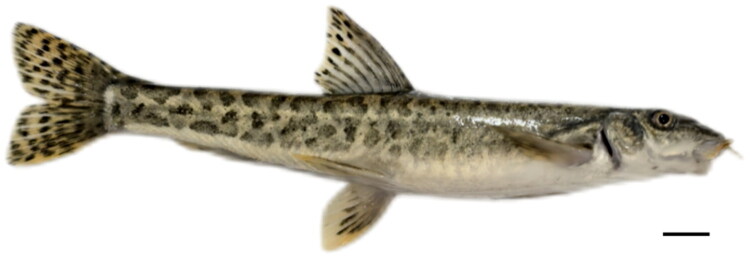
Reference image of *Triplophysa scleroptera*. The specimen shown is the individual used for mitochondrial genome sequencing (catalog number: LSU-2024-05-0116). The scale bar represents 1 cm. Photograph was taken by the author of this article, Fei Li.

In May 2024, live specimens of *T. scleroptera* were collected from the Yellow River, Tianzhu Tibetan Autonomous County, Gansu Province, China (36°18′41.82″N, 103°25′02.40″E), where species-level identification of *T. scleroptera* was achieved using established nemacheilid taxonomic keys for *Triplophysa* loaches (Wu and Wu 1992). Notable identification features of *T. scleroptera* include an elongate, scaleless body with a complete lateral line, three pairs of rostral and maxillary barbels surrounding an inferior, papillose mouth, and dorsal- and anal-fin ray counts. Subsequent to collection, digital imaging of specimens was performed using a Nikon D850 digital SLR system (Tokyo, Japan) prior to humane euthanasia through eugenol overdose administration. Post-procedural tissue sampling involved the excision of muscle tissue from imaged specimens, which were immediately stored in 95% ethanol for molecular preservation. The remaining specimens underwent complete fixation in 95% ethanol prior to archival deposition at the Industrial College of Traditional Chinese Medicine and Health (Lishui University). These accessioned specimens have been formally assigned the catalog number LSU-2024-05-0116 (Figure 1), with Dr. Jie Chen (jchen@lsu.edu.cn) designated as the contact person.

Genomic DNA isolation was performed on muscle samples employing the Rapid Animal Genomic DNA Isolation Kit (Sangon Biotech, Shanghai, China), following manufacturer’s protocols. DNA libraries with a 350-bp insert size were constructed using the TruSeq Nano™ kit (Illumina, San Diego, CA) and sequenced on the Illumina NovaSeq X Plus platform, generating 150-bp paired-end reads. Quality control and adapter trimming were performed using Fastp v0.20.0 (Chen et al. 2018), filtered paired-end reads that were used for mitochondrial genome reconstruction. Quality-filtered reads were first mapped to the *Triplophysa tenuis* reference mitogenome (GenBank: KT224363) using BWA-MEM v0.7.17 (Li 2013). Mapped reads were assembled with SPAdes v4.10 (Prjibelski et al. 2020; k-mer: 21,33,55), generating a 16,572 bp draft contig. Crucially, the identical read set was independently subjected to *de novo* assembly using GetOrganelle v1.7.7 (Jin et al. 2020) with extended k-mer ranges (21–127), yielding an identical 16,572 bp contig that contained all 37 canonical mitochondrial elements. This congruence confirmed absence of reference bias and sample contamination. The consensus contig was circularized using MitoZ v2.4 (Meng et al. 2019), polished twice with Pilon v1.24 (Walker et al. 2014) for error correction, and annotated through a multi-validation pipeline: structural predictions from MITOS2 (Donath et al. 2019) and MiTFi (Jühling et al. 2012) were cross-verified via homology searches (NCBI BLAST+ v2.28; Camacho et al. 2009), gene prediction (GeneWise; Birney et al. 2004), and structural RNA detection (Infernal v1.1; Nawrocki and Eddy 2013). All annotations were manually curated in Geneious Prime v2024.0.7 (Geneious 2025). Coverage depth was validated by realigning raw reads to the final assembly (Bowtie2 v2.3.4; Langmead and Salzberg 2012), with per-base depth calculated via SAMtools v1.16.1 (Li et al. 2009) and visualized using ggplot2 (Wickham 2016; Fig. S1).

For phylogenetic reconstruction, we included 26 sampled *Triplophysa* species and used *Aborichthys elongatus* as the outgroup. The analysis based on the concatenated nucleotide sequences of the 13 mitochondrial PCGs was performed to evaluate the phylogenetic position of *T. scleroptera* among the sampled *Triplophysa* taxa. The 13 PCGs were extracted using PhyloSuite v1.2.3 (Zhang et al. 2020) from *T. scleroptera* and the selected comparative taxa. The nucleotide sequences of each PCG were aligned separately in codon mode using MAFFT v7.388 (Katoh and Standley 2013), and the aligned genes were then concatenated into a combined dataset. Phylogenetic reconstruction was conducted using the maximum-likelihood method implemented in IQ-TREE v2.2.0 (Minh et al. 2020). The concatenated dataset was initially partitioned into 39 data blocks corresponding to the three codon positions of each of the 13 PCGs, and the optimal partition scheme and substitution models were selected using ModelFinder (Kalyaanamoorthy et al. 2017). Nodal support was assessed with 1000 bootstrap replicates. The resulting phylogenetic tree was visualized and annotated using the Interactive Tree of Life platform (iTOL v6) (Letunic and Bork 2021).

## Results

**Figure 2.**
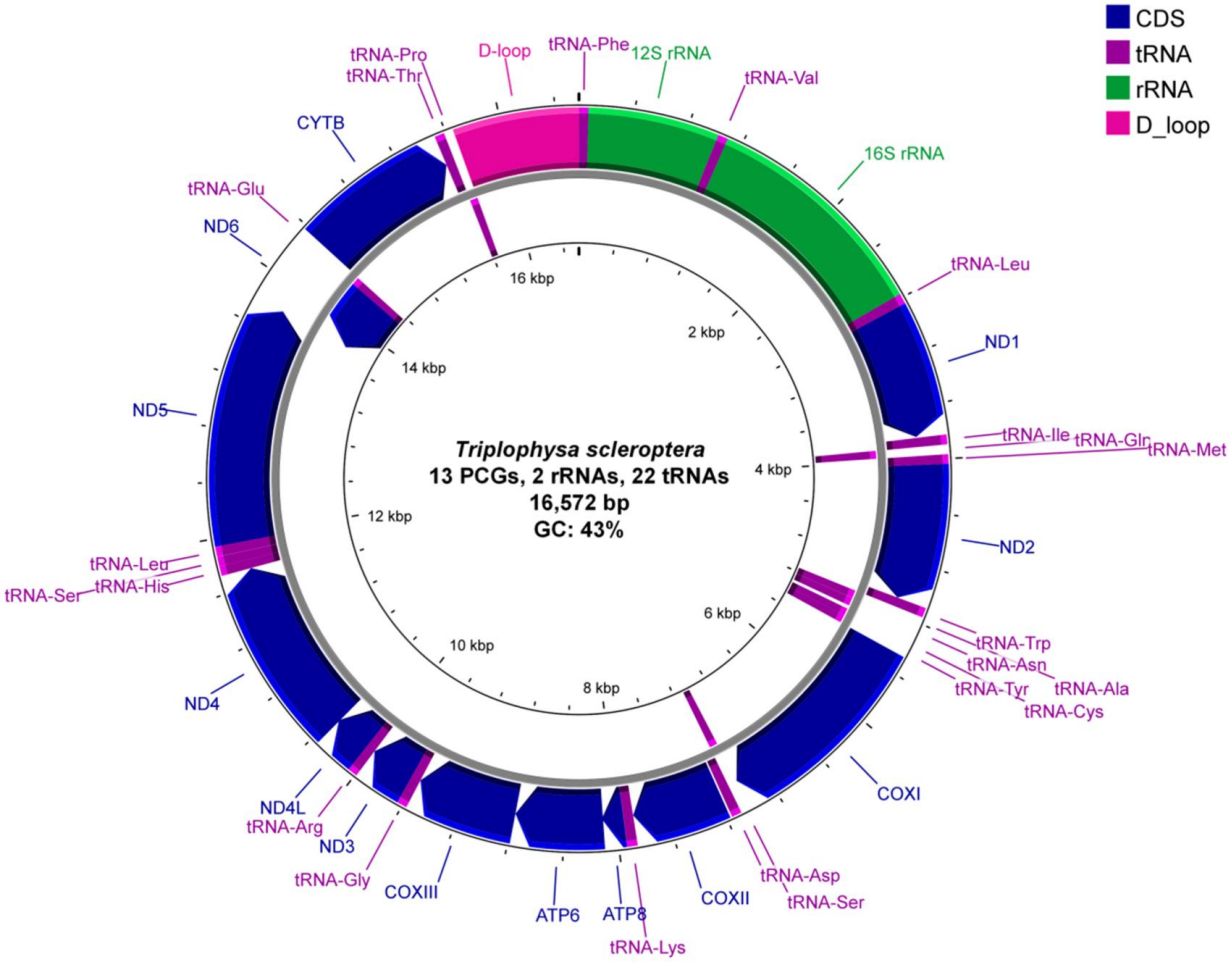
Circular map of the mitochondrial genome of *Triplophysa scleroptera*. Genes are depicted on their transcribed strand, with arrows indicating transcriptional direction.

The mitochondrial architecture of *T. scleroptera* exhibited characteristic conservation, presenting a circular 16,572 bp genome organized into 37 functional elements: 13 PCGs, 22 transfer RNA genes, two ribosomal RNA genes, and a non-coding regulatory domain. Detailed characteristics of the 13 mitochondrial PCGs are summarized in Table S1. Nucleotide distribution analysis revealed base composition asymmetry (A: 28.24%, T: 28.58%, G: 17.82%, and C: 25.36%) with an aggregate GC-content of 43%. Strand-specific gene distribution showed H-strand encoding 28 elements (12 PCGs, 14 tRNA genes, and rRNA clusters), while L-strand harbored nine genetic elements (*ND6* PCG + eight tRNA genes). Using the vertebrate mitochondrial code (translation table 2), most PCGs start with ATG, whereas *COX1* start with GTG. Termination patterns comprised complete (TAA/TAG) and incomplete stop codons, the latter observed in *ND2*, *COX2*, *COX3*, *ND3*, *ND4*, and *CYTB* genes requiring polyadenylation for functional completion. tRNA gene lengths ranged from 66 to 76 bp, whereas ribosomal RNA genes showed length differences (*rrnL* = 1678 bp; *rrnS* = 951 bp) and distinct GC profiles (43.09% vs. 49.21%). A 917-bp non-coding region—putatively corresponding to the displacement loop (D-loop)—was detected between *trnF* (tRNA-Phe) and *trnP* (tRNA-Pro); its GC content is 34.13% (Figure 2).

**Figure 3.**
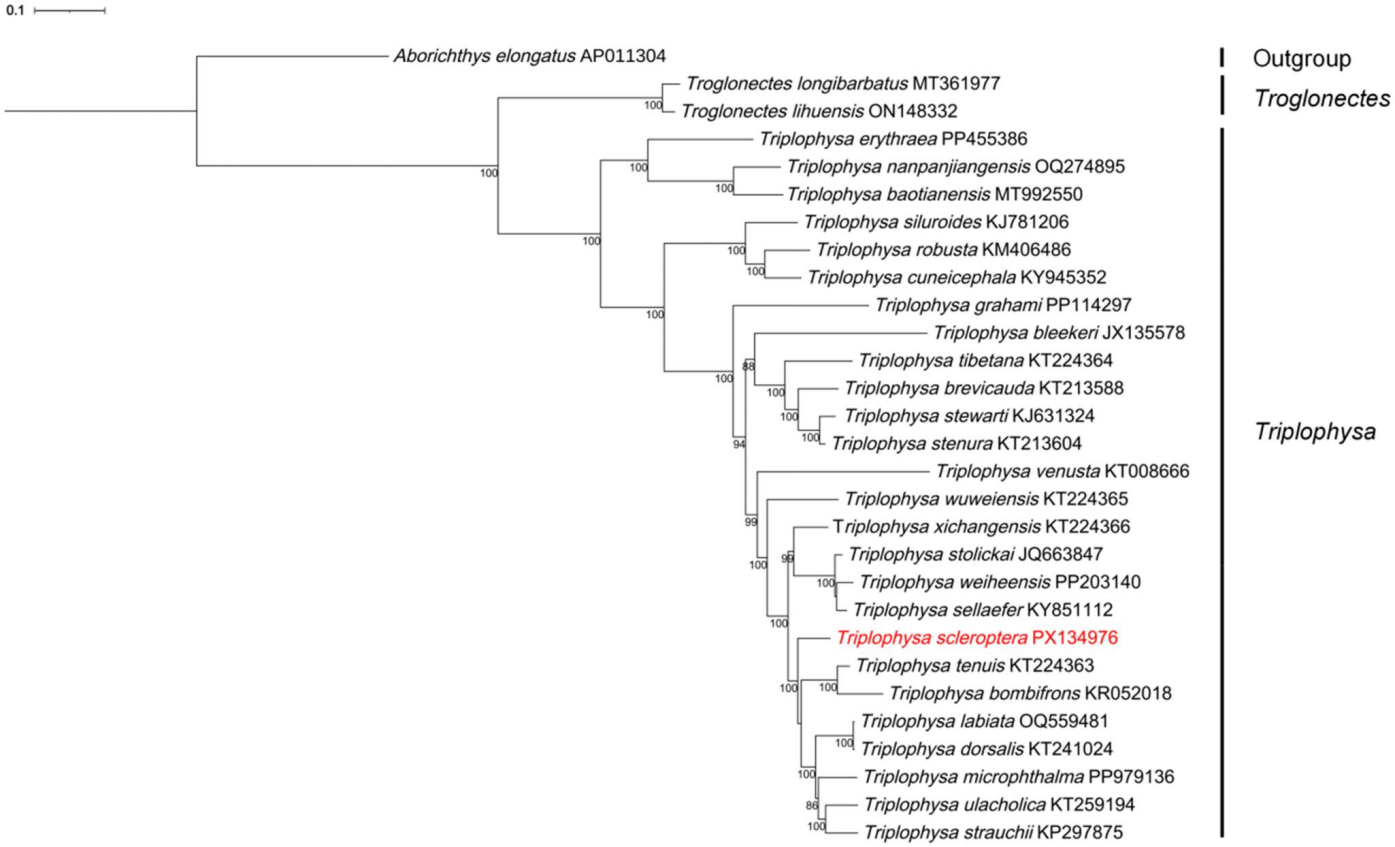
Maximum-likelihood phylogenetic tree inferred from the concatenated nucleotide sequences of the 13 mitochondrial protein-coding genes (PCGs) of *Triplophysa scleroptera* and representative species of Nemacheilidae. Bootstrap support values (>70%) are shown above the branches. *Aborichthys elongatus* (AP011304) was used as the outgroup taxa. The mitogenome analyzed in this study, *T. scleroptera* (PX134976), is highlighted in red. Detailed information for all sequences used in the phylogenetic analysis is provided in Supplementary Table S2.

The maximum-likelihood phylogenetic tree based on the concatenated nucleotide sequences of 13 mitochondrial PCGs showed that *T. scleroptera* clustered within the sampled *Triplophysa* taxa. *A. elongatus* was used as the outgroup to root the tree. Within the sampled *Triplophysa* species, *T. scleroptera* was recovered as sister to the clade formed by *T. tenuis* and *T. bombifrons*. Most major nodes were supported by high bootstrap values (Figure 3).

## Discussion and conclusions

The complete mitochondrial genome of *T. scleroptera* shows the canonical teleost architecture (37 genes plus a control region on a 16.5 kb circular molecule) with slight A + T bias and the typical distribution of genes between strands, closely resembling other *Triplophysa* mitogenomes (e.g. *T. dorsalis*, *T. bombifrons*, *T. strauchii*, *T. nanpanjiangensis*, and *T. grahami*) (Lei et al. 2016; Zhao et al. 2023; Xu et al. 2024). The presence of alternative start codons and incomplete stop codons is also common in teleost mitogenomes and reflects conserved translational mechanisms rather than species-specific features (Saitoh et al. 2000).

The control region of *T. scleroptera* is of moderate length and flanked by tRNA genes, a configuration shared with other nemacheilid loaches (Lei et al. 2016; Zhao et al. 2023). Given that the D-loop is usually the most variable part of the mitogenome, this region of *T. scleroptera* is likely to provide informative markers for population structure and phylogeography in the upper Yellow River drainage.

Phylogenetic analysis based on concatenated mitochondrial PCGs recovered a *Triplophysa* clade among the sampled taxa, in agreement with previous mitogenomic studies on plateau loaches (Wang et al. 2016, 2023b). Within this clade, *T. scleroptera* was placed as sister to the clade formed by *T. tenuis* and *T. bombifrons*. This result provides additional comparative mitogenomic information for the sampled *Triplophysa* taxa.

In summary, the mitogenome of *T. scleroptera* enriches the genomic resources available for Nemacheilidae and offers a robust framework for future work on taxonomy, population genetics, and high-altitude adaptation of plateau loaches. When combined with denser taxon sampling and nuclear genomic data, these results will contribute to a more comprehensive understanding of the origin and diversification of Tibetan and adjacent riverine fish faunas.

## Supplementary Material

ARRIVE checklist.docx

Figure S1.doc

Table S2.docx

Table S1.docx

## Data Availability

The genome sequence data supporting this study are openly available in GenBank of NCBI at https://www.ncbi.nlm.nih.gov under the accession number PX134976. The associated BioProject, SRA, and BioSample numbers are PRJNA1300846, SRR34847608, and SAMN50429082, respectively.

## References

[CIT0001] Birney E, Clamp M, Durbin R. 2004. GeneWise and Genomewise. Genome Res. 14(5):988–995. 10.1101/gr.1865504 15123596 PMC479130

[CIT0002] Camacho C et al. 2009. BLAST+: architecture and applications. BMC Bioinformatics. 10(1):421. 10.1186/1471-2105-10-421 20003500 PMC2803857

[CIT0003] Chen IS, Liu GD, Prokofiev AM. 2016. The complete mitochondrial genome of giant stone loach *Triplophysa siluroides* (Cypriniformes: Balitoridae). Mitochondrial DNA A DNA Mapp Seq Anal. 27(2):998–1000. 10.3109/19401736.2014.926523 24963763

[CIT0004] Chen S, Zhou Y, Chen Y, Gu J. 2018. Fastp: an ultra-fast all-in-one FASTQ preprocessor. Bioinformatics. 34(17):i884–i890. 10.1093/bioinformatics/bty560 30423086 PMC6129281

[CIT0005] Chen W, Yang J, Li Y, Li X. 2019. Exploring taxonomic diversity and biogeography of the family Nemacheilinae (Cypriniformes). Ecol Evol. 9(18):10343–10353. 10.1002/ece3.5553 31624553 PMC6787813

[CIT0006] Donath A et al. 2019. Improved annotation of protein-coding genes boundaries in metazoan mitochondrial genomes. Nucleic Acids Res. 47(20):10543–10552. 10.1093/nar/gkz833 31584075 PMC6847864

[CIT0007] Feng X et al. 2025. Chromosome-level genome assembly of *Triplophysa scleroptera*. Sci Data. 12(1):1082. 10.1038/s41597-025-05122-5 40595745 PMC12218405

[CIT0008] Feng X, Chen Y, Sui X, Chen Y. 2019. The complete mitochondrial genome of *Triplophysa cuneicephala* (Cypriniformes: Balitoridae) with phylogenetic consideration. Mitochondrial DNA B Resour. 4(1):1239–1240. 10.1080/23802359.2019.1591245

[CIT8606930] Geneious. 2025. Geneious Prime v2024.0.7. https://www.geneious.com.

[CIT0009] Jin JJ et al. 2020. GetOrganelle: a fast and versatile toolkit for accurate de novo assembly of organelle genomes. Genome Biol. 21(1):241. 10.1186/s13059-020-02154-5 32912315 PMC7488116

[CIT0010] Jühling F et al. 2012. Improved systematic tRNA gene annotation allows new insights into the evolution of mitochondrial tRNA structures and into the mechanisms of mitochondrial genome rearrangements. Nucleic Acids Res. 40(7):2833–2845. 10.1093/nar/gkr1131 22139921 PMC3326299

[CIT0011] Kalyaanamoorthy S, Minh BQ, Wong TK, Von Haeseler A, Jermiin LS. 2017. ModelFinder: fast model selection for accurate phylogenetic estimates. Nat Methods. 14(6):587–589. 10.1038/nmeth.4285 28481363 PMC5453245

[CIT0012] Katoh K, Standley DM. 2013. MAFFT multiple sequence alignment software version 7: improvements in performance and usability. Mol Biol Evol. 30(4):772–780. 10.1093/molbev/mst010 23329690 PMC3603318

[CIT0013] Langmead B, Salzberg SL. 2012. Fast gapped-read alignment with Bowtie 2. Nat Methods. 9(4):357–359. 10.1038/nmeth.1923 22388286 PMC3322381

[CIT0014] Lei D et al. 2016. The complete mtDNA genome of *Triplophysa dorsalis* (Cypriniformes, Balitoridae, Cobitoidea): genome characterization and phylogenetic analysis. Mitochondrial DNA A DNA Mapp Seq Anal. 27(5):3745–3746. 10.3109/19401736.2015.1079886 26457606

[CIT0015] Letunic I, Bork P. 2021. Interactive Tree Of Life (iTOL) v5: an online tool for phylogenetic tree display and annotation. Nucleic Acids Res. 49(W1):W293–W296. 10.1093/nar/gkab301 33885785 PMC8265157

[CIT0016] Li H et al. 2009. The sequence alignment/map format and SAMtools. Bioinformatics. 25(16):2078–2079. 10.1093/bioinformatics/btp352 19505943 PMC2723002

[CIT0017] Li H. 2013. Aligning sequence reads, clone sequences and assembly contigs with BWA-MEM. arXiv 1303.3997.

[CIT0018] Li J, Si S, Guo R, Wang Y, Song Z. 2013. Complete mitochondrial genome of the stone loach, *Triplophysa stoliczkae* (Teleostei: Cypriniformes: Balitoridae). Mitochondrial DNA. 24(1):8–10. 10.3109/19401736.2012.710225 22920274

[CIT0019] Li P, Liu J, Wang T, Wang J. 2023. Estimates of the age, growth, and mortality of *Triplophysa scleroptera* (Herzenstein, 1888) in the upper reaches of the Yellow River, China. Fishes. 8(9):457. 10.3390/fishes8090457

[CIT0020] Meng G, Li Y, Yang C, Liu S. 2019. MitoZ: a toolkit for animal mitochondrial genome assembly, annotation and visualization. Nucleic Acids Res. 47(11):e63. 10.1093/nar/gkz173 30864657 PMC6582343

[CIT0021] Minh BQ et al. 2020. IQ-TREE 2: new models and efficient methods for phylogenetic inference in the genomic era. Mol Biol Evol. 37(5):1530–1534. 10.1093/molbev/msaa015 32011700 PMC7182206

[CIT0022] Nawrocki EP, Eddy SR. 2013. Infernal 1.1: 100-fold faster RNA homology searches. Bioinformatics. 29(22):2933–2935. 10.1093/bioinformatics/btt509 24008419 PMC3810854

[CIT0023] Niu YM, Liu SJ, Tian F, Feng CG, Zhao K. 2024. Mitochondrial genome characteristics and phylogenetic analysis of *Triplophysa weiheensis*. Genomics Appl Biol. 43(Z2):1813–1825.

[CIT0024] Prjibelski A, Antipov D, Meleshko D, Lapidus A, Korobeynikov A. 2020. Using SPAdes de novo assembler. Curr Protoc Bioinformatics. 70(1):e102. 10.1002/cpbi.102 32559359

[CIT0025] Rashid J et al. 2025. Identification of a Nemacheilid loach from Kaptai Lake, Bangladesh using morphology and two molecular markers. Ecol Genet Genom. 34:100318. 10.1016/j.egg.2024.100318

[CIT0026] Saitoh K et al. 2000. Complete nucleotide sequence of Japanese flounder (*Paralichthys olivaceus*) mitochondrial genome: structural properties and cue for resolving teleostean relationships. J Hered. 91(4):271–278. 10.1093/jhered/91.4.271 10912672

[CIT0027] Tang Q et al. 2013. The complete mitochondrial genome sequence of *Triplophysa bleekeri* (Teleostei, Balitoridae, Nemacheilinae). Mitochondrial DNA. 24(1):25–27. 10.3109/19401736.2012.716050 22947117

[CIT0028] Walker BJ et al. 2014. Pilon: an integrated tool for comprehensive microbial variant detection and genome assembly improvement. PLOS One. 9(11):e112963. 10.1371/journal.pone.0112963 25409509 PMC4237348

[CIT0029] Wang C et al. 2023a. A comprehensive analysis of *Triplophysa labiata* (Kessler, 1874) mitogenome and its phylogenetic implications within the *Triplophysa* genus. Genes. 14(7):1356. 10.3390/genes14071356 37510261 PMC10378854

[CIT0030] Wang J et al. 2019. The complete mitochondrial genome of *Triplophysa tibetana*. Mitochondrial DNA B Resour. 4(1):1411–1412. 10.1080/23802359.2019.1598297

[CIT0031] Wang X et al. 2023b. Complete mitogenome of *Triplophysa bombifrons*: comparative analysis and phylogenetic relationships among the members of *Triplophysa*. Genes. 14(1):128. 10.3390/genes14010128 36672869 PMC9858811

[CIT0032] Wang Y et al. 2016. Mitogenomic perspectives on the origin of Tibetan loaches and their adaptation to high altitude. Sci Rep. 6(1):29690. 10.1038/srep29690 27417983 PMC4945904

[CIT0033] Wang Y et al. 2021. The complete mitochondrial genome of a cave-dwelling loach *Triplophysa baotianensis* (Teleostei: Nemacheilidae). Mitochondrial DNA B Resour. 6(3):1209–1211. 10.1080/23802359.2021.1899861 33829087 PMC8008877

[CIT0034] Wang YD, Dong XY, Liu HB. 2024. The complete mitochondrial genome of *Triplophysa erythraea* (Huang et al. 2019) (Cypriniformes, Nemacheilidae). Mitochondrial DNA B Resour. 9(10):1424–1428. 10.1080/23802359.2024.2417939 39450203 PMC11500520

[CIT0035] Wickham H. 2016. ggplot2: elegant graphics for data analysis. Springer. 10.1007/978-3-319-24277-4_9

[CIT0036] Wu YF, Wu CZ. 1992. Fishes of the Qinghai-Tibetan Plateau. Sichuan Science and Technology Press.

[CIT0037] Xu M, Zhang J, Song J, Zhang Z, Wu J. 2024. The complete mitochondrial genome of *Triplophysa grahami* Regan 1906 (Cypriniformes: Nemacheilidae) and phylogenetic analysis. Mitochondrial DNA B Resour. 9(9):1190–1195. 10.1080/23802359.2024.2399926 39247499 PMC11378653

[CIT0038] Yan P, Li J, Ma Q, Deng Y, Song Z. 2016. Complete mitochondrial genome of *Triplophysa robusta* (Teleostei: Cypriniformes: Balitoridae). Mitochondrial DNA A DNA Mapp Seq Anal. 27(3):1715–1716. 10.3109/19401736.2014.961136 25238109

[CIT0039] Yan Y, Luo D. 2016. The complete mitochondrial genome sequence of *Triplophysa stenura* (Teleostei, Cypriniformes): genome characterization and phylogenetic analysis. Mitochondrial DNA B Resour. 1(1):607–608. 10.1080/23802359.2016.1209093 33490413 PMC7800979

[CIT0040] Yang P et al. 2024. Characterization and phylogenetic analysis of the complete mitochondrial genome of *Triplophysa microphthalma*. Biology. 13(8):608. 10.3390/biology13080608 39194546 PMC11351504

[CIT0041] Zhang D et al. 2020. PhyloSuite: an integrated and scalable desktop platform for streamlined molecular sequence data management and evolutionary phylogenetics studies. Mol Ecol Resour. 20(1):348–355. 10.1111/1755-0998.13096 31599058

[CIT0042] Zhao J et al. 2023. The complete mitochondrial genome of *Triplophysa nanpanjiangensis* Zhu and Cao 1988 (Cypriniformes: Nemacheilidae). Mitochondrial DNA B Resour. 8(12):1360–1363. 10.1080/23802359.2023.2290119 38196794 PMC10776044

