# Supplementary material for: The complete mitochondrial genome of Triplophysa scleroptera and its phylogenetic placement among related nemacheilid taxa
Source: Mitochondrial DNA B Resour. 2026 May 10;11(6):722–6. doi: 10.1080/23802359.2026.2668246 (PMC13162541; doi:10.1080/23802359.2026.2668246)
Supplement: ARRIVE checklist.docx [file TMDN_A_2668246_SM4194.docx]

**ARRIVE 2.0 Author Checklist (Completed)**

Manuscript: “The complete mitochondrial genome of Triplophysa scleroptera (Cypriniformes: Nemacheilidae) and its phylogenetic analysis”

| **ARRIVE item (2.0)** | **What the guideline asks you to report (brief)** | **Where reported in your manuscript / reason if not reported** |
| --- | --- | --- |
| 1. Study design (Essential 10) | Describe the study design, groups compared (incl. controls) and the experimental unit. | Single-specimen field collection + sequencing/annotation + phylogenetic reconstruction (no intervention groups). Experimental unit: one individual / one mitogenome assembly. |
| 2. Sample size (Essential 10) | State exact n for each group; explain how sample size was decided (incl. a priori calculation, if done). | Number of individuals used for sequencing in this study (n=1)/No samples excluded |
| 3. Inclusion/exclusion criteria (Essential 10) | State inclusion/exclusion criteria for animals/units and data; report exclusions and n per analysis. | Not reported; typically not applicable for descriptive mitogenome assembly. No samples or data points were excluded. |
| 4. Randomisation (Essential 10) | State whether randomisation was used; describe methods and confounder minimisation. | Not applicable (no allocation to treatment groups). |
| 5. Blinding (Essential 10) | State who was blinded to group allocation at different stages. | Not applicable (no group allocation). |
| 6. Outcome measures (Essential 10) | Define all outcome measures; specify primary outcome for hypothesis-testing studies. | Outcome measures: mitogenome length, gene content/features; phylogenetic placement and bootstrap support (Results/Figures). No primary outcome (not hypothesis-testing). |
| 7. Statistical methods (Essential 10) | Describe statistical methods; assumptions; effect sizes/precision where relevant. | Phylogenetic inference: ML in IQ-TREE with model selection and bootstrap replication (Materials and methods: phylogenetic reconstruction). No other statistical testing. |
| 8. Experimental animals (Essential 10) | Provide species/strain, sex, age/developmental stage, weight (as relevant), provenance/health status. | Species and collection site reported; sex/age/weight not stated (field-caught fish). |
| 9. Experimental procedures (Essential 10) | Describe procedures in enough detail (what, how, when, where, why), including anaesthesia/euthanasia and sample collection. | Collection details, imaging, euthanasia via eugenol overdose, tissue sampling and preservation reported in Materials and methods (first paragraph). |
| 10. Results (Essential 10) | Provide summary statistics and measures of variability/precision; report n; describe exclusions. | Genome features and phylogenetic results reported. |
| 11. Abstract (Recommended set) | Accurate summary including species, key methods, principal findings and conclusions. | Abstract includes species, mitogenome length/components, and phylogenetic conclusion. |
| 12. Background (Recommended set) | Provide sufficient scientific background and rationale. | Introduction provides family/genus context, need for mitogenome data, and rationale. |
| 13. Objectives (Recommended set) | Clearly state objectives and/or hypotheses. | Stated at end of Introduction (determine mitogenome; reconstruct ML phylogeny). |
| 14. Ethical statement (Recommended set) | Provide ethical review/approval, licence details and justifications where relevant. | Ethical approval section includes committee approval number and welfare compliance; ARRIVE adherence statement present. |
| 15. Housing and husbandry (Recommended set) | Describe housing, husbandry and environmental enrichment (if animals were housed). | Not applicable (wild-caught; no housing reported). |
| 16. Animal care and monitoring (Recommended set) | Describe analgesia, monitoring, humane endpoints, adverse events. | Euthanasia method reported; No adverse events observed. |
| 17. Interpretation/scientific implications (Recommended set) | Interpret results in the context of objectives, limitations and literature. | Discussion interprets genome characteristics and phylogenetic placement; suggest future work. |
| 18. Generalisability/translation (Recommended set) | Comment on generalisability/translation (as relevant). | Briefly discussed as providing resources for taxonomy/population genetics and future nuclear data integration. |
| 19. Protocol registration (Recommended set) | State whether a protocol was registered; provide where. | Not reported; Not registered. |
| 20. Data access (Recommended set) | Provide data availability, accession numbers and where data/code can be accessed. | GenBank accession and BioProject/SRA/BioSample provided in Data availability statement. |
| 21. Declaration of interests (Recommended set) | Declare conflicts of interest and funding sources; role of funders if relevant. | Disclosure statement and Funding section provided. |
