# Supplementary material for: The complete mitochondrial genome of Triplophysa scleroptera and its phylogenetic placement among related nemacheilid taxa
Source: Mitochondrial DNA B Resour. 2026 May 10;11(6):722–6. doi: 10.1080/23802359.2026.2668246 (PMC13162541; doi:10.1080/23802359.2026.2668246)
Supplement: Figure S1.doc [file TMDN_A_2668246_SM4193.doc]

**The complete mitochondrial genome of *Triplophysa scleroptera* (Cypriniformes: Nemacheilidae) and its phylogenetic analysis**

Fei Li, Zengxiang Guo, Qiang Hu, Yan Pan, Weijun Wu, Jie Chen


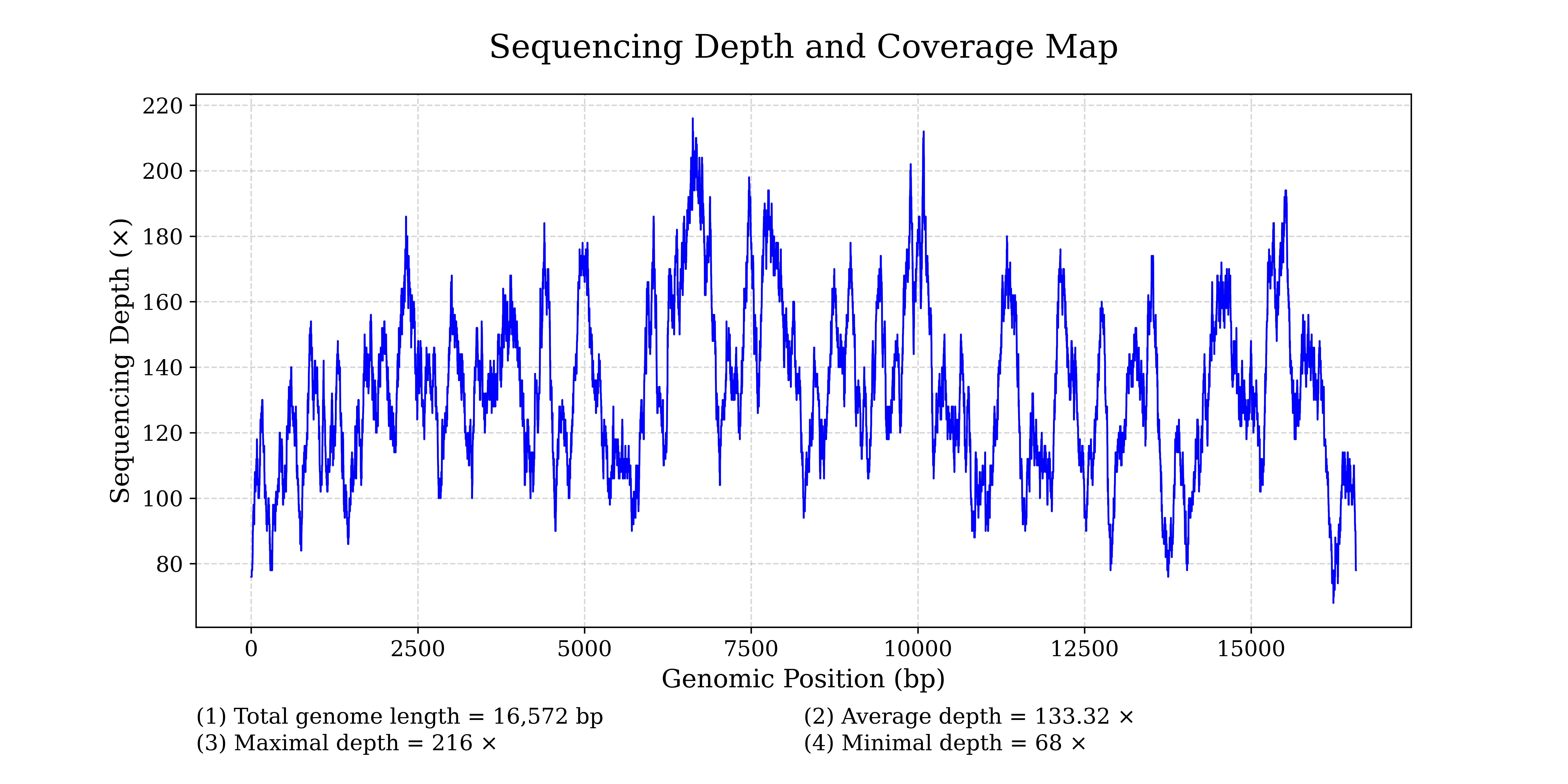


**Figure S1.** Depth of coverage for *Triplophysa scleroptera* mitochondrial genome. X and Y axis present nucleotide position of *T. scleroptera* mitochondrial genome and coverage depth, respectively.
