# Supplementary material for: The complete mitochondrial genome of Triplophysa scleroptera and its phylogenetic placement among related nemacheilid taxa
Source: Mitochondrial DNA B Resour. 2026 May 10;11(6):722–6. doi: 10.1080/23802359.2026.2668246 (PMC13162541; doi:10.1080/23802359.2026.2668246)
Supplement: Table S2.docx [file TMDN_A_2668246_SM4192.docx]

**Table S2.** Mitochondrial genomes used for phylogenetic analysis.

| No. | Taxon | Family/subfamily | GenBank accession | Sequence length (bp) | Reference |
| --- | --- | --- | --- | --- | --- |
| 1 | *Aborichthys elongatus* | Nemacheilidae | AP011304 | 16,544 | unpublished |
| 2 | *Troglonectes longibarbatus* | Nemacheilidae | MT361977 | 16,569 | unpublished |
| 3 | *Troglonectes lihuensis* | Nemacheilidae | ON148332 | 16,568 | unpublished |
| 4 | *Triplophysa erythraea* | Nemacheilidae | PP455386 | 16,565 | Wang et al., 2024 |
| 5 | *Triplophysa nanpanjiangensis* | Nemacheilidae | OQ274895 | 16,558 | Zhao et al., 2023 |
| 6 | *Triplophysa baotianensis* | Nemacheilidae | MT992550 | 16,576 | Wang et al., 2021 |
| 7 | *Triplophysa siluroides* | Nemacheilidae | KJ781206 | 16,574 | Chen et al., 2016 |
| 8 | *Triplophysa robusta* | Nemacheilidae | KM406486 | 16,570 | Yan et al., 2016 |
| 9 | *Triplophysa cuneicephala* | Nemacheilidae | KY945352 | 16,571 | Feng et al., 2019 |
| 10 | *Triplophysa grahami* | Nemacheilidae | PP114297 | 16,566 | Xu et al., 2024 |
| 11 | *Triplophysa bleekeri* | Nemacheilidae | JX135578 | 16,568 | Tang et al., 2013 |
| 12 | *Triplophysa tibetana* | Nemacheilidae | KT224364 | 16,574 | Wang et al., 2016 |
| 13 | *Triplophysa brevicauda* | Nemacheilidae | KT213588 | 16,572 | Wang et al., 2016 |
| 14 | *Triplophysa stewarti* | Nemacheilidae | KJ631324 | 16,567 | unpublished |
| 15 | *Triplophysa stenura* | Nemacheilidae | KT213604 | 16,569 | Yan and Luo, 2016 |
| 16 | *Triplophysa venusta* | Nemacheilidae | KT008666 | 16,574 | Wang et al., 2016 |
| 17 | *Triplophysa wuweiensis* | Nemacheilidae | KT224365 | 16,681 | Wang et al., 2016 |
| 18 | *Triplophysa xichangensis* | Nemacheilidae | KT224366 | 16,570 | Wang et al., 2016 |
| 19 | *Triplophysa stoliczkai* | Nemacheilidae | JQ663847 | 16,571 | Li et al., 2013 |
| 20 | *Triplophysa weiheensis* | Nemacheilidae | PP203140 | 16,570 | Niu et al., 2024 |
| 21 | *Triplophysa sellaefer* | Nemacheilidae | KY851112 | 16,571 | Feng et al., 2019 |
| 22 | *Triplophysa scleroptera* | Nemacheilidae | PX134976 | 16,572 | This study |
| 23 | *Triplophysa tenuis* | Nemacheilidae | KT224363 | 16,571 | Wang et al., 2016 |
| 24 | *Triplophysa bombifrons* | Nemacheilidae | KR052018 | 16,569 | Han et al., 2016 |
| 25 | *Triplophysa strauchii* | Nemacheilidae | KP297875 | 16,590 | Han et al., 2016 |
| 26 | *Triplophysa labiata* | Nemacheilidae | OQ559481 | 16,573 | Wang et al., 2023 |
| 27 | *Triplophysa dorsalis* | Nemacheilidae | KT241024 | 16,572 | Lei et al., 2016 |
| 28 | *Triplophysa microphthalma* | Nemacheilidae | PP979136 | 16,569 | Yang et al., 2024 |
| 29 | *Triplophysa ulacholica* | Nemacheilidae | KT259194 | 16,568 | Wang et al., 2016 |
