# Supplementary material for: The complete mitochondrial genome of Triplophysa scleroptera and its phylogenetic placement among related nemacheilid taxa
Source: Mitochondrial DNA B Resour. 2026 May 10;11(6):722–6. doi: 10.1080/23802359.2026.2668246 (PMC13162541; doi:10.1080/23802359.2026.2668246)
Supplement: Table S1.docx [file TMDN_A_2668246_SM4191.docx]

**Table S1.** Organization of the 13 mitochondrial protein-coding genes (PCGs) of *Triplophysa scleroptera*.

| **Gene** | **From** | **To** | **Length (bp)** | **Strand** | **5' spacer/overlap**  **(bp)** | **Start codon** | **Stop codon** | **3' spacer/overlap**  **(bp)** |
| --- | --- | --- | --- | --- | --- | --- | --- | --- |
| ND1 | 2,777 | 3,751 | 975 | H | 0 | ATG | TAA | 7 |
| ND2 | 3,969 | 5,015 | 1,047 | H | 0 | ATG | T-- | -2 |
| COX1 | 5,396 | 6,946 | 1,551 | H | 1 | GTG | TAA | 0 |
| COX2 | 7,106 | 7,810 | 705 | H | 13 | ATG | T-- | -14 |
| ATP8 | 7,874 | 8,041 | 168 | H | 1 | ATG | TAA | -10 |
| ATP6 | 8,032 | 8,715 | 684 | H | -10 | ATG | TAA | -1 |
| COX3 | 8,715 | 9,515 | 801 | H | -1 | ATG | T-- | -17 |
| ND3 | 9,572 | 9,922 | 351 | H | 0 | ATG | T-- | -2 |
| ND4L | 9,991 | 10,287 | 297 | H | 0 | ATG | TAA | -7 |
| ND4 | 10,281 | 11,663 | 1,383 | H | -7 | ATG | T-- | -1 |
| ND5 | 11,876 | 13,714 | 1,839 | H | 0 | ATG | TAG | -4 |
| ND6 | 13,711 | 14,232 | 522 | L | -4 | ATG | TAA | 0 |
| CYTB | 14,307 | 15,467 | 1,161 | H | 5 | ATG | T-- | -20 |

Negative values indicate overlapping nucleotides between adjacent genes, whereas positive values indicate intergenic spacers. T-- indicates an incomplete stop codon.
